# Oncogenic H-Ras Expression Induces Fatty Acid Profile Changes in Human Fibroblasts and Extracellular Vesicles

**DOI:** 10.3390/ijms19113515

**Published:** 2018-11-08

**Authors:** Krizia Sagini, Lorena Urbanelli, Eva Costanzi, Nico Mitro, Donatella Caruso, Carla Emiliani, Sandra Buratta

**Affiliations:** 1 Department of Chemistry, Biology and Biotechnology, University of Perugia, 06123 Perugia, Italy; krizia.sagini@studenti.unipg.it (K.S.); lorena.urbanelli@unipg.it (L.U.); eva.costanzi@studenti.unipg.it (E.C.); carla.emiliani@unipg.it (C.E.); 2 Department of Pharmacological and Biomolecular Sciences, University of Milan, 20133 Milan, Italy; nico.mitro@unimi.it (N.M.); donatella.caruso@unimi.it (D.C.); 3 CEMIN-Center of Excellence for Innovative Nanostructured Material, 06123 Perugia, Italy

**Keywords:** extracellular vesicles, oncogene-induced senescence, H-Ras, fatty acids, desaturases, elongases, acyl-coenzyme A synthetases

## Abstract

Extracellular vesicles (EVs) are lipid bilayer surrounded particles that are considered an additional way to transmit signals outside the cell. Lipids have not only a structural role in the organization of EVs membrane bilayer, but they also represent a source of lipid mediators that may act on target cells. Senescent cells are characterized by a permanent arrest of cell proliferation, but they are still metabolically active and influence nearby tissue secreting specific signaling mediators, including those carried by EVs. Notably, cellular senescence is associated with increased EVs release. Here, we used gas chromatography coupled to mass spectrometry to investigate the total fatty acid content of EVs released by fibroblasts undergoing H-RasV12-induced senescence and their parental cells. We find that H-RasV12 fibroblasts show increased level of monounsaturated and decreased level of saturated fatty acids, as compared to control cells. These changes are associated with transcriptional up-regulation of specific fatty acid-metabolizing enzymes. The EVs released by both controls and senescent fibroblasts show a higher level of saturated and polyunsaturated species, as compared to parental cells. Considering that fibroblasts undergoing H-RasV12-induced senescence release a higher number of EVs, these findings indicate that senescent cells release via EVs a higher amount of fatty acids, and in particular of polyunsaturated and saturated fatty acids, as compared to control cells.

## 1. Introduction

Extracellular vesicles (EVs) have been implicated in many physiological processes [1]. The first observation considered EVs as a cellular mean to discard unneeded material during cell differentiation [2]. Later, it emerged that EVs transmit signals and they are now considered an alternative manner of cell signal transmission [3,4]. Three main types of EVs have been described, i.e., microvesicles budding from the plasma membrane (100–1000 nm), exosomes originating from the inward budding of late endosomes (30–150 nm), and apoptotic bodies released by cells undergoing apoptosis [5]. Despite their apparent simple classification, their similar and overlapping biochemical properties make it difficult to obtain preparation containing exclusively microvesicles or exosomes, so EVs are preferentially indicated as small EVs and large EVs, enriched either in exosomes or microvesicles, respectively [6]. EVs have been found in every fluid of the body, including easily accessible ones such as saliva or discard products such as urine. For this reason, and because EVs often maintain important properties of the cells secreting them, EVs have gained considerable attention for diagnostic and therapeutic purposes [7]. EVs contain lipids, proteins and nucleic acids, namely ncRNA such as miRNAs and lncRNA and a few databases have been developed, such as EVpedia [8], listing the biochemical components retrieved in EVs. 

Despite their relevance, the number of studies on EV lipid composition is limited as compared to studies on protein or nucleic acid composition [9,10,11,12]. There is a certain consensus that EVs are enriched in cholesterol, sphingomyelin, ether-linked phospholipids and lysophospholipids [12,13]. EVs contain not only lipids forming the bulk of their membranes, but also lipid mediators acting as carriers between cells [14,15]. Studies have shown the presence within EVs of lipid mediators and enzymes involved in the release of their precursors [16], demonstrating that EVs may be important players in the so-called “transcellular biosynthesis” of eicosanoids [17,18]. Fatty acid analyses provide evidence that EVs mainly contain saturated fatty acids (SFA), even if monounsaturated (MUFA) and polyunsaturated (PUFA) ones are also present. In fact, EVs can be also considered an additional mechanism to transport fatty acids across the plasma membrane to target cells [19]. Thus, understanding the fatty acid composition of EVs may allow us to identify what kind of fatty acids are preferentially discarded by cells, influencing the metabolism of the surrounding tissue [20].

Senescence is a condition characterized by a permanent arrest of cell proliferation [21,22]. Senescent cells are metabolically active and release in the extracellular environment specific biochemical mediators, collectively known as Senescence Associated Secretory Phenotype (SASP) [23]. SASP can deeply influence surrounding cells, namely through the action of cytokines and chemokines stimulating the clearance of senescent cells by innate immune system and maintaining autocrine senescence signals. Although the absence of proliferation by senescent cells is considered a barrier towards oncogenic transformation [24,25,26], many SASP factors also have pro-tumorigenic properties [27] and EVs are currently considered additional SASP components [28]. 

Oncogene-induced senescence (OIS) is due to the activation of oncogenes in normal cells in the absence of additional oncogenic activation and/or inactivation of tumor suppressor genes that are necessary to fully transform cells. OIS is phenotypically indistinguishable from cellular senescence and cells undergoing are characterized by flattened morphology and positivity to well established senescence markers, namely as senescence-associated β-galactosidase [29]. The Ras family of oncogenes encodes small monomeric GTP-binding proteins that transduce mitogenic stimuli. It is well established that expression of constitutively active H-RasV12 induces senescence [30]. We have previously demonstrated that fibroblasts undergoing H-RasV12-induced senescence release a higher number of EVs [12], in agreement with studies on other senescence models [31,32]. Further, EVs released by fibroblasts undergoing H-RasV12-induced senescence show a peculiar lipid signature, as they are enriched in lysosphospholipids, ether-linked lipids and sulfatides [12]. Nevertheless, the total fatty acid composition of EVs released by OIS cells is not known, although it is a key assessment to understand what kind of fatty acids are preferentially discarded by the cells via EVs and how they influence the metabolism of neighbouring cells.

Here, we investigate the total fatty acid composition of EVs released by H-RasV12 and control fibroblasts, and their parental cells. Our findings provide evidence that EVs released by senescent and control fibroblasts share a similar fatty acid profile, which is enriched in SFA and PUFA as compared to releasing cells. Taking into account that senescent fibroblasts release a higher number of EVs, these findings indicate that senescent cells release a higher amount of fatty acids, in particular SFA and PUFA, with respect to controls. Besides this, we observed a significant increase of MUFA in cells undergoing senescence, in association with changes in the expression profile of enzymes involved in fatty acid desaturation and release from membrane phospholipids.

## 2. Results

### 2.1. Analysis of H-RasV12 and Control Fibroblasts Released EVs by Immunoblotting and Immuno-TEM

**Figure 1:**
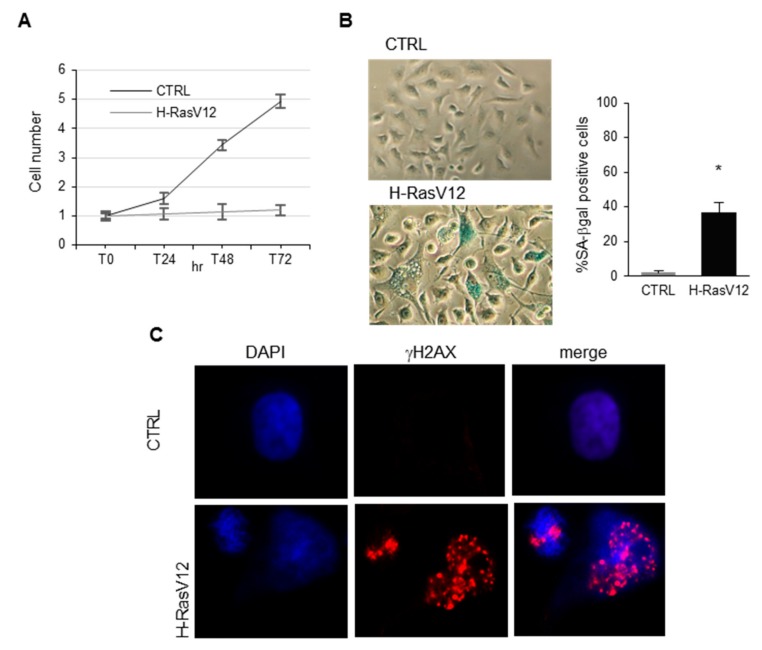

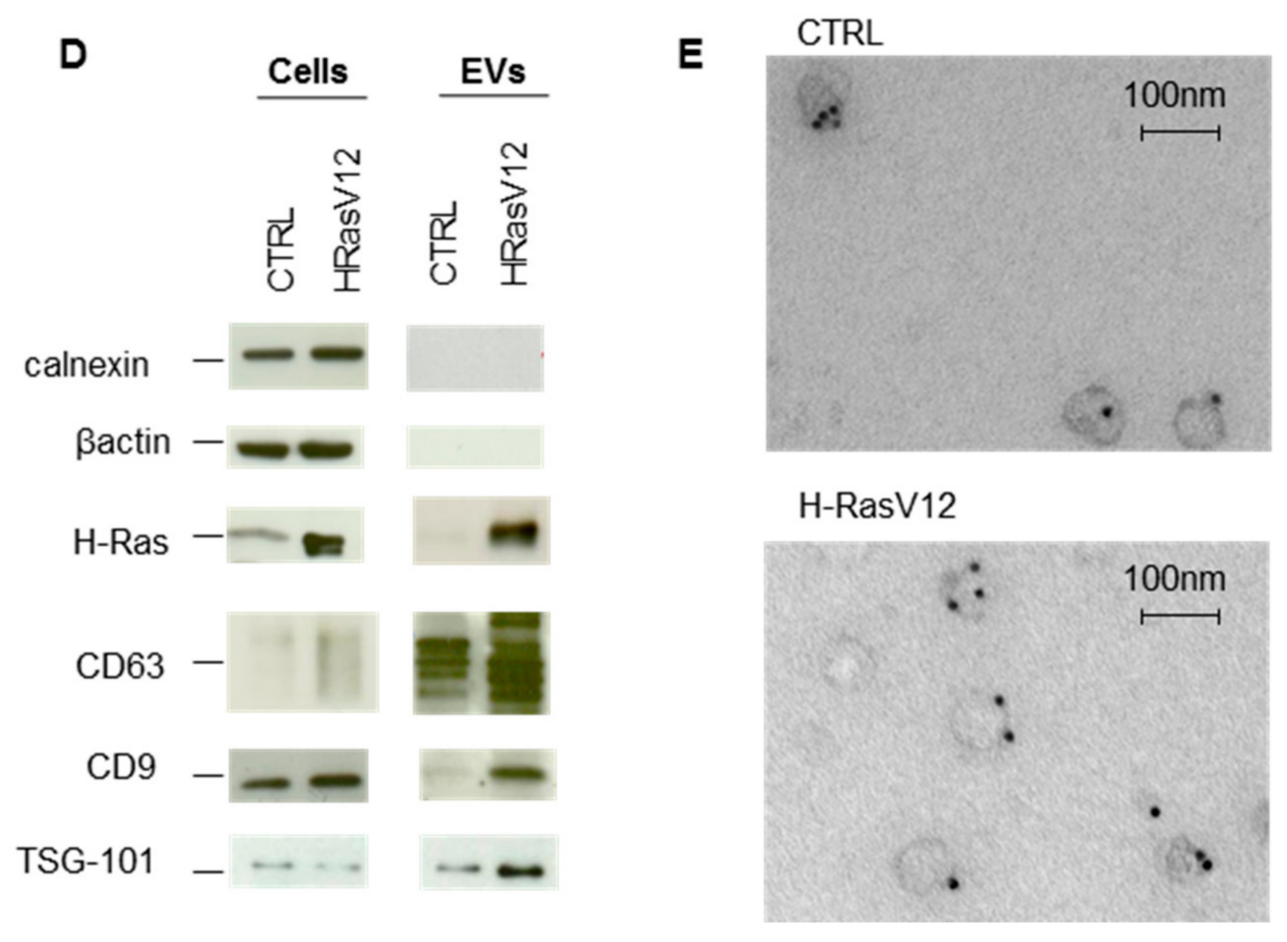
Analysis of H-RasV12-induced senescence in HuDe fibroblasts and characterization of EVs released by control and H-RasV12 expressing cells. (**A**) Growth curve of HuDe fibroblasts expressing H-RasV12 or transfected with the vector alone as control (CTRL). Mean values were calculated on 3 replicates and mean ± S.E.M. is indicated as fold increase with respect to the number of seeded cells (set 1). (**B**) Senescence-associated β-galactosidase staining and quantification of senescence-associated β-galactosidase (SA-β-gal) positive cells. Microscopy images (40×) of H-RasV12 and CTRL fibroblasts transfected with the vector alone. SA-β-gal positive cells were counted on three different fields in three separate experiments experiments (* *p* < 0.05, CTRL vs. H-RasV12). (**C**) Immunostaining for γH2AX. Cells were fixed in 4% paraformaldehyde, permeabilized in PBS/0.1% Triton X-100, incubated with an anti-γH2AX and labelled with an anti-rabbit Alexa-Fluor 594 antibody. Nuclei were stained with 1 µg/mL DAPI. Fluorescence microscopy analysis was carried out with a Nikon TE2000 microscope through a 60× oil immersion objective. (**D**) Immunoblotting. Cell extracts and EVs samples were separated by SDS-PAGE, electrotransferred, and probed with positive and negative markers indicated. (**E**) Immuno-transmission electron micrographs of EVs. Samples were fixed, dropped directly onto formvar/carbon coated grids, blocked and incubated with mouse anti-CD63 primary antibody, rabbit anti-mouse secondary antibody and gold-labelled Protein A.

H-RasV12 was expressed in HuDe fibroblasts by transfection and cells were pharmacologically selected with blasticidin-S to get rid of untransfected cells. pcDNA6 empty vector was transfected as control. H-RasV12 expression induced an arrest of cell proliferation, changes in cell morphology accompanied by senescence-associated β-galactosidase staining and DNA damage demonstrated by γH2AX immunoreactivity (Figure 1). H-RasV12 expression was checked by immunoblotting with anti-H-Ras antibody (Figure 1). EVs were isolated from fibroblasts expressing H-RasV12 and control cells using the polymer co-precipitation method (Exoquick-TC was used as reagent) and their main features characterized as previously described [12]. Immunoblotting and immuno-TEM analysis showed the presence in our preparations of proteins specifically enriched in EVs (Figure 1). Positive markers CD9, CD63 and Tsg101 were clearly detectable in EVs, in agreement with guidelines [5]. Calnexin, an endoplasmic reticulum protein usually not present in EVs, and actin were used as negative markers. As expected, they were not detectable in EVs, although they were clearly present in equal amount in cell samples (Figure 1). In addition, H-RasV12 was clearly present in EVs released by fibroblasts over-expressing it.

The structural characterization of EVs was carried out by immuno-TEM (Figure 1). Image analysis detected small EVs of less than 100 nm size in H-RasV12 and control samples, compatible with an enrichment in small EVs. The presence of CD63 on their membrane bilayer was confirmed using immunogold labelling with an anti-CD63. These results confirmed an enrichment of small membranous vesicles in our preparation, consisting of exosomes and small microvesicles [12].

### 2.2. Analysis of Fatty Acids Content

**Figure 2:**
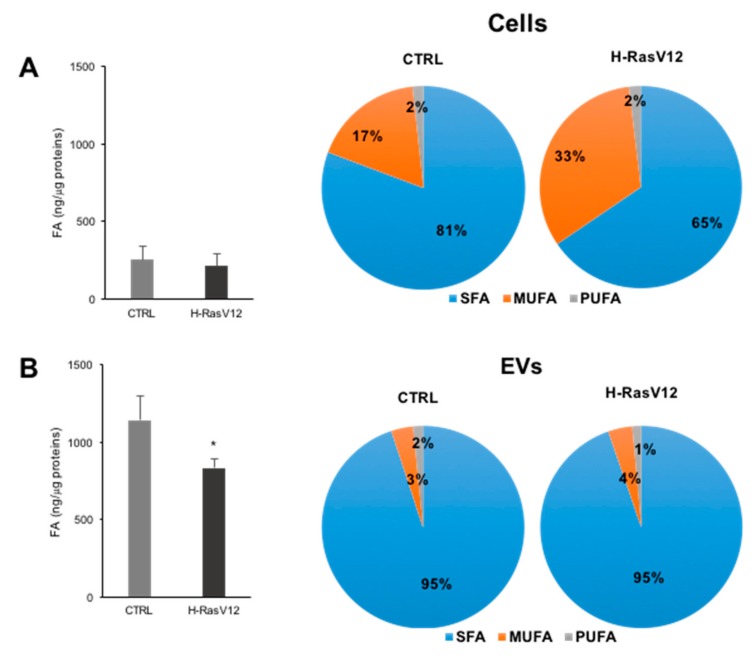
Fatty acid content and distribution of SFA, MUFA and PUFA in control and H-RasV12 cells (**A**) and their released EVs (**B**). Lipids were extracted and total fatty acids analysis was carried out by GC-MS. In the graphs are reported the amounts of total fatty acids relative to protein content. Data are expressed as ng of FA/µg of proteins and are presented as mean ± SD (*n* = 6) (* *p* < 0.05, control vs. H-RasV12). In the pie charts are reported the proportion of fatty acids grouped on the basis of their unsaturation level; SFA: saturated fatty acids; MUFA: Mono-unsaturated fatty acids; PUFA: Poly-unsaturated fatty acids.

The GC-MS analysis of fatty acids in both cells and EVs highlighted significant differences between cells and EVs (Figure 2). First, EVs had a higher fatty acids/protein ratio with respect to cells (Figure 2A,B) and the content of total fatty acids normalized for proteins was lower for EVs prepared from H-RasV12 cells as compared to controls (Figure 2B). The high lipid/protein ratio in EVs with respect to cells agrees with previous studies [10,22,33]. In addition, when we grouped fatty acids in saturated (SFA), monounsaturated (MUFA) and polyunsaturated (PUFA), we clearly observed that H-RasV12 expressing fibroblasts were enriched in MUFA (~33% of the total detected fatty acids as compared to 17% of control samples) (Figure 2A). This increase was associated with the decrease of SFA (~65% of the total detected fatty acids as compared to 80% of control), whereas the content of PUFA was similar. EVs were characterised by a similar and elevated SFA level in both samples (Figure 2B), which is consistent with previous studies [9,10].

**Figure 3:**
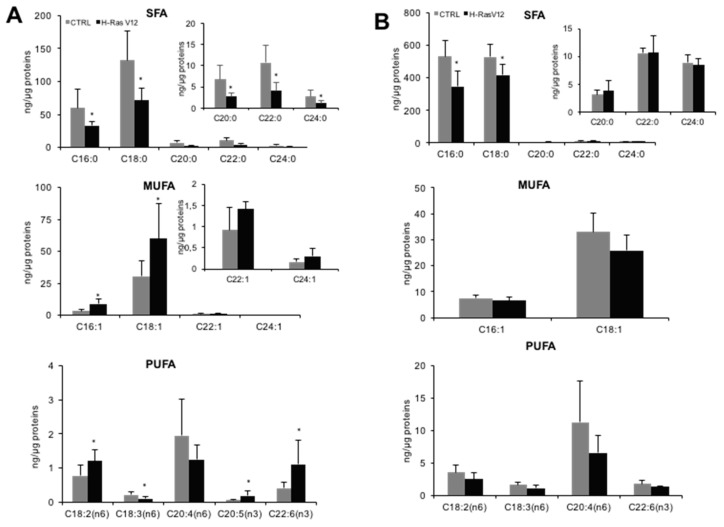
Fatty acid profiles of control and H-RasV12 expressing fibroslasts (**A**) and their released EVs (**B**). Lipids were extracted, and total fatty acids composition were analysed by GC-MS. Data, are expressed as ng fatty acids/µg proteins and are presented as mean ± S.D (*n* = 6). * *p* < 0.05 (control vs. H-RasV12). SFA: saturated fatty acids; MUFA: mono-unsaturated fatty acids; PUFA: poly-unsaturated fatty acids.

When the fatty acids profile was analyzed in detail (Figure 3A), significant modifications were observed in cells undergoing H-RasV12-inducing senescence. The most relevant ones were the significant decrease of all SFA species and the significant increase of palmitoleic (C16:1) and oleic (C18:1) acids, leading to a general increase of MUFA in senescent cells. Regarding PUFA, in H-RasV12 fibroblasts we observed decreased levels of γ-linolenic acid (C18:3 n6) and an increased level of γ-linoleic (C18:2 n6), docosahexaenoic (C22:6 n3) and eicosapentaenoic (C20:5 n3) acids (Figure 3A). 

The comparison between cells and EVs revealed that the higher fatty acids/protein ratio in EVs with respect to cells was due to a high SFA and PUFA content (Table 1). As for fibroblasts, palmitic (C16:0) and stearic (C18:0) acids were the most abundant species in EVs (Figure 3B). Besides, comparison of MUFA species in cells and in EVs revealed that two monounsaturated species, C22:1 and C24:1, were not detectable in EVs (Figure 3). As mentioned above, a greater amount of PUFA in EVs with respect to parental cells was observed. In fact, even if eicosapentaenoic acid was not detected in EVs, they were enriched in each PUFA with respect to parental cells (Figure 3B).

The comparison of the composition of EVs released from H-RasV12 and control fibroblasts revealed a significant decrease of the two most abundant SFA species (C16:0 and C18:0) that was in line with the decrease in ng of total fatty acids/µg of proteins observed for H-RasV12 EVs (Figure 2B, left). No additional changes in the levels of MUFA and PUFA species were observed (Figure 3B), thus indicating the H-RasV12 expressing cells release EVs with a similar fatty acid profile as compared to control cells.

### 2.3. Expression Analysis of Genes Involved in Fatty Acids Remodelling and Phospholipases A2

**Figure 4:**
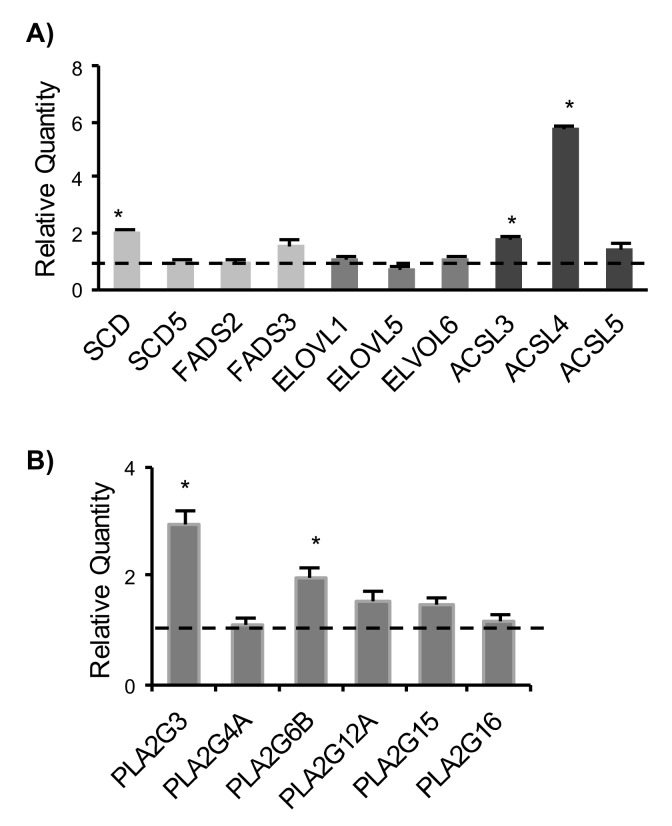
Gene expression analysis of fatty acid-metabolizing enzymes in control and H-RasV12 expressing fibroblasts by qRT-PCR. (**A**) Gene expression analysis of desaturases (*SCD* and *FADS*), elongases (*ELOVL*) and acyl-coenzyme A synthetases (*ACSL*). (**B**) Gene expression analysis of phospholipases A2 (*PLA2*). Ten ng of each cDNA were used as template. Reactions were performed in triplicate, using SYBR green technology StepOne RT-PCR machine to detect amplification. The GAPDH gene was used as endogenous control. The fold expression in H-RasV12 fibroblasts with respect to control is displayed, expressed as Relative Quantity (RQ). The analysis was repeated three times in triplicate and the mean ± S.D. is reported (* *p* < 0.05).

To gain insight into transcriptional changes underlying the different fatty acid profile of H-RasV12 vs. control fibroblasts, we analyzed the expression of several genes involved in fatty acid metabolism, i.e., desaturases (stearoyl-CoA desaturase, *SCD* and fatty acids desaturase, *FADS*), elongases (*ELOVL*) and acyl-coenzyme A synthetases (*ACSL*) by qRT-PCR. Among genes expressed in fibroblasts (Supplementary Table S1), we observed an up-regulation of *SCD* and notably, of 2 out of 3 *ACSL* genes, i.e., *ACSL3* and *ACSL4* (Figure 4A). The increased expression of *SCD* gene (stearoyl-CoA desaturase 1 or delta-9 desaturase) in H-RasV12 fibroblasts (Figure 4A) well correlated with the observation that our model of oncogene-induced senescence is characterized by higher content of C16:1 and C18:1 (Figure 3A). Furthermore, we investigated the expression of phospholipase A2 transcripts, which are involved in the release of fatty acid precursor of lipid mediators from membrane phospholipid and found that *PL2G3* and *PLA2G6B* were significantly up-regulated (Figure 4B).

## 3. Discussion

Extracellular vesicles (EVs) have been recognized as an additional way to transmit cell-to-cell signals. Much evidence has been also provided that their lipid content not only has a structural function, but that they can also represent conveyors of membrane-derived bioactive lipids [14,15]. Analysis of fatty acids released extracellularly via EVs is an important factor in the determination of the amount and type of lipid mediator precursors available to the surrounding tissue and to extracellular enzymes, in order to spread lipid-mediated signals. To gain insight on how H-RasV12 expression in human fibroblasts influences fatty composition of cells and EVs, we have investigated the fatty acid profile of H-RasV12 and control fibroblasts and their released EVs.

Fatty acid composition confirmed that EVs have a higher fatty acid content per µg of protein respect to parental cells. This is consistent with other studies that have shown a higher lipid to protein ratio for EVs with respect to the releasing cells [10,12]. Lipid/protein ratio has been also proposed as a marker for EVs [33]. Grouping fatty acids into SFA, MUFA and PUFA category showed that H-RasV12 fibroblasts are characterized by a significantly increased level of MUFA and a decreased level of SFA as compared to control cells, whereas the content of PUFA was similar. 

The level of SFA, MUFA and PUFA in EVs revealed that they were mostly made up of saturated species (about 95%). This was consistent with previous studies [9,10]. Comparing fatty acid content between cells and EVs, we observed an enrichment of PUFA in EVs, as well as SFA, whereas MUFA were present at a similar level. PUFA are precursor of lipid mediators which can have either a pro-inflammatory or a pro-resolving function [34]. Their presence in EVs has been previously described, as well as the presence of enzymes responsible for lipid mediator biosynthesis, such as prostaglandins [16,18,35]. Considering that fibroblasts undergoing H-RasV12-induced senescence release a higher number of EVs [12], these findings indicate that senescent cells release via EVs a higher amount of fatty acids, and in particular of PUFA and SFA, as compared to control cells. As discussed above, PUFA availability could affect the biosynthesis of lipid mediators in recipient cells following EVs uptake. As for SFA, their increased availability could represent an alternative source of fatty acids for recipient cells in neighbor tissue. This might be relevant for tissue homeostasis, as it has been previously shown that SFA-enriched EVs may induce pro-inflammatory responses [36,37].

From a structural point of view, the high level of SFA, and saturated phospholipid species, has been previously correlated with an increased membrane rigidity relative to parent cell membranes [9,10,38,39] and with an increased stability of EVs in biological fluids [40].

In fibroblasts, the increased level of MUFA in cells undergoing H-RasV12-induced senescence was mainly due to an increase of palmitoleic acid (C16:1) and oleic acid (C18:1), whereas their corresponding saturated fatty acids palmitic (C16:0) and stearic (C18:0) were decreased. The conversion of C16:0 and C18:0 in C16:1 and C18:1 is due to stearoyl-CoA desaturase (*SCD*), that introduces a cis-Δ9 double bond into saturated fatty acids. Gene expression analysis showed a significant up-regulation of *SCD* gene, whereas the other desaturases were not affected. The conversion of palmitic acid (C16:0) into stearic acid (C18:0), which was the most abundant species, requires the activity of elongases, but none of the expressed elongases showed a significantly altered transcript level. We also tested the expression of fatty acid activating enzymes acyl CoA synthetases, which are key enzymes converting long-chain fatty acids into fatty acyl-CoA esters, which then serve as a substrate for both lipid synthesis and β-oxidation [41]. We detected a significantly increased level of two enzymes, *ACSL3* and *ACSL4*. Even if these two enzymes catalyze the same reaction, there is increasing evidence for a specialization in the substrates. In fact, *ACSL3* prefers palmitate and *ACSL4* arachidonic acid (C20:4) and eicosapentaenoic acid (C20:5), thus modulating prostaglandin E(2) release from human smooth muscle cells [42]. These evidences suggest that H-RasV12-induced senescence may be associated with pro-inflammatory fatty acid metabolism. *ACSL3* and *ACSL4* expression levels have been reported to be frequently altered in cancer [43] but here we provide evidence that they have also an altered expression profile in senescence induced by oncogenic H-Ras. This finding is consistent with previous studies, demonstrating the up-regulation of *ACSL3* in NeuT oncogene-induced senescence [44] and *ACSL4* in BJ fibroblasts undergoing replicative senescence [45].

The first step of fatty acid release from membrane (to make lipid mediator precursors available for the biosynthetic reactions) is the hydrolysis of fatty acids from the sn-2 position of phospholipids by phospholipase A2, to give free fatty acids and lysophospholipids [46]. We tested a panel of human phospholipase A2 and detected an up-regulation of *PL2G3* and *PLA2G6B*. *PLA2G3* encodes for a secreted phospholipase A2 [47]. *PLA2G3* has a relevant role in male reproduction and is an endogenous regulator of mast cells [48], but no role in H-Ras induced senescence has not been reported so far. *PLA2GB6B* encodes for an isoform of inducible phospholipase A2 also known as iPLA2γ. This enzyme promotes cellular membrane hydrolysis and prostaglandin production [49], reinforcing the evidence that H-RasV12 induced senescence may be associated with an increased release of pro-inflammatory lipid mediator precursors such as arachidonic acid.

Lipid metabolism changes induced by H-Ras activation have been often investigated with a specific attention on intracellular metabolic alterations. Here, we provide evidence that H-RasV12-induced senescence is associated with changes in fatty acids content and in the level of some fatty acid metabolizing enzymes transcripts. Whether these changes are a specific feature of oncogene induced senescence or are associated with senescence induced by other types of stimuli such as telomere shortening or DNA damage remains to be determined. However, the changes in the expression profile of the fatty acids-related enzymes have an impact not only on cellular lipid composition, but also on the levels of saturated and polyunsaturated fatty acids released extracellularly via EVs. These fatty acids represent a factor that contributes to the properties of the senescence-associated secretory phenotype, influencing metabolism and cell signaling to nearby tissue.

## 4. Materials and Methods

### 4.1. HuDe Fibroblasts Transfection and H-RasV12 Expression

HuDe (human dermal fibroblasts) were purchased from the Istituto Zooprofilattico Sperimentale, Brescia, Italy as previously reported [25]. Cells were cultured in DMEM (Dulbecco’s modified Eagle’s medium) with 10% (*v*/*v*) heat-inactivated FBS, 2 mM glutamine, 100 U/mL penicillin, 100 mg/mL streptomycin at 37 °C in a 5% CO_2_. Cell viability was assessed by trypan blue staining exclusion (0.1% in 0.9% NaCl) and cell growth was determined by counting cell numbers in a hemocytometer. Cells were transiently transfected with the pcDNA6 plasmid encoding the constitutively active mutant H-RasV12 and with the empty vector as control using Lipofectamine LTX, then selected for 5 to 7 days with 4 µg/mL blasticidin-S as selective agent, as previously described [12]. 

### 4.2. Staining of Senescence Associated β-Galactosidase (SA-β-Gal)

Cells were washed with PBS, fixed with 0.5% glutaraldehyde for 10 min at RT, washed with PBS and incubated at 37 °C overnight with the staining solution (5 mM potassium ferrocyanide, 5 mM potassium ferricyanide, 2 mM MgCl_2_ in 100 mM citric acid and 200 mM Na_2_HPO_4_ solutions pH 6.0, 1 mg/mL 5-bromo-4-chloro-3-indolyl β-d-galactopyranoside). The development of blue color was checked under microscope using 20× total magnification. Positive fibroblasts were counted and results expressed as mean ± S.E.M. of the percentage of SA-β-gal positive fibroblasts with respect to the total number of fibroblasts.

### 4.3. Immunostaining

Cells were washed with PBS, fixed in 4% paraformaldehyde for 20 min at RT, permeabilized with 0.1% Triton X-100 in PBS for 10 min at RT, then incubated with an anti-γH2AX (Santa Cruz Biotechnology, Santa Cruz, CA, USA) in 2% FBS/0.01% Triton X-100/PBS and labelled with an anti-rabbit Alexa-Fluor 594 antibody (Thermofisher Scientific, Waltham, MA, USA). Nuclei were stained with 1 μg/mL DAPI (Sigma-Aldrich, St. Louis, MO, USA). Fluorescence microscopy analysis was carried out using a Nikon TE2000 microscope (Minato, Tokyo, Japan) through a 60× oil immersion objective.

### 4.4. Extracellular Vesicles Preparation

After selection with 4 µg/mL blasticidin-S, HuDe fibroblasts were further incubated for 72 h in serum free medium containing 4 µg/mL blasticidin-S to avoid any contamination by FBS lipoproteins. Then, medium was collected and centrifuged to remove cells, cell debris and large EVs (300× *g*, 10 min; 2000× *g*, 10 min), adding a filtration step at 0.22 µm with cellulose filter (Millipore, Burlington, MA, USA) to enrich for small EVs. Vesicles were isolated by polymer co-precipitation using Exoquick-TC precipitation method (System Biosciences, Palo Alto, CA, USA) as previously described [12]. Pelleted EVs were stored at −80 °C in PBS. EV protein content was determined by the Bradford method, using bovine serum albumin as standard.

### 4.5. Immunoblotting

Cell extracts were prepared as previously described [12]. Cell extracts (30 μg) or EVs (3 µg) were mixed with sample buffer 5× (1M Tris-HCl pH 6.8, 5% (*w*/*v*) SDS, 6% (*v*/*v*) glycerol, 0.01% (*w*/*v*) Bromophenol blue) without DTT (non-reducing conditions, used for CD9 and CD63 antibodies) or with 125 mM DTT (other antibodies). Samples were electrophoresed on 12% acrylamide gel at 150 V for 1 h and transferred to PVDF membrane at 100 V for 1 h. As internal control, actin or calnexin were used. Rabbit polyclonal anti-H-Ras antibody, mouse monoclonal anti-Tsg101 antibody, goat polyclonal anti-calnexin antibody were from Santa Cruz Biotechnology, mouse monoclonal anti-CD9 and mouse monoclonal anti-CD63 were from Abcam (Cambridge, UK), mouse monoclonal anti β-actin was from Sigma-Aldrich. Sheep anti-goat (Sigma), donkey anti-rabbit and sheep anti-mouse HRP-linked secondary antibodies (Sigma Aldrich) were probed according to manufacturer’s instructions. Immunoblots were detected by chemiluminescence using ECL system.

### 4.6. Immunogold

For immuno-transmission electron microscopy (immuno-TEM), EVs were fixed in 4% formaldehyde and 0.1% glutaraldehyde for 15 min then dropped directly onto formvar/carbon coated grids and left for 20 min at room temperature. Grids were washed with PBS, blocked for 10 min with 0.5% bovine serum albumin in PBS, incubated for 20 min with mouse anti-CD63 primary antibody, incubated 20 min with rabbit anti-mouse secondary antibody and, finally, incubated with gold-labelled protein A for 10 min. Between each antibody incubation, grids were washed twice with PBS. Two washes, one in PBS and one in water, were performed before grids stained/embedded with 0.4% uranyl acetate/1.8% methyl cellulose for 10 min at 4 °C. Grids were allowed to dry at least for 20 min and samples were observed on a JEOL-JEM 1230 at 80 kV and images were recorded with a Morada digital camera.

### 4.7. Cells and Vesicles Preparation for Fatty Acid Analysis

For fatty acids analysis, about 3.6 × 10^6^ of H-RasV12 expressing cells or control cells were washed twice with PBS, pelleted and stored at −80 °C. Total cellular lipids were extracted from 6 cell pellets from 3 different preparations and protein concentration was determined in each sample to normalize lipid content. For EVs, they were obtained from cell culture medium of the same preparations used for cell lipid extraction, pooled, and 8 µg of proteins used for each analysis. Lipid extraction was carried out as previously reported [50,51]. Extracts were dried under nitrogen and resuspended in methanol prior to be submitted for analyses. An acid hydrolysis was carried out by dissolving the methanol fraction in chloroform/methanol 1:1 (*v*/*v*). Then, 1 M HCl:methanol (1:1, *v*/*v*) solution was added to the total lipid extract and the mixed solution shaken for 2 h. Chloroform:water (1:1 *v*/*v*) was added and the organic phase collected and dried under nitrogen flow. The residue was dissolved in methanol. For fatty acid quantification, the MS analysis was carried out with a selective ion monitoring-tandem mass spectrometry (SIM-MS/MS) method. Quantitative analysis was performed with calibration curves. An ESI source connected with an API 4000 triple quadrupole instrument (AB Sciex, Old Connecticut Path, Framingham, MA USA) was used. The mobile phases were: Water/10 mM isopropylethylamine/15 mM acetic acid (phase A) and methanol (phase B). MultiQuant™ software version 3.0.2 (AB Sciex, Old Connecticut Path, Framingham, MA, USA) was used for data analysis and peak review of chromatograms. Quantitative evaluation of phospholipid families was performed based on standard curves. Quantitative data were normalized on the protein content of cells or vesicles. An external standard for each phospholipid family was used for the semi-quantitative analysis. Data were normalized on protein content.

### 4.8. qRT-PCR

RNA was extracted and retro-transcribed as previously described [12]. cDNA was used to determine the expression of genes listed in Supplementary Table S1. cDNA was used to determine transcript levels by qRT-PCR in a StepOne RT-PCR machine (Applied Biosystems, Foster City, CA, USA) using SYBR^®^ Select Master Mix (Life Technologies, Carlsbad, CA, USA). Reactions were performed in triplicate and GAPDH used as endogenous control. Data were analysed using the ΔΔ*C*t method. Δ*C*t was calculated subtracting the average *C*t value of *GAPDH* to the average *C*t value of a specific gene for each sample, then ΔΔ*C*t as the difference between the Δ*C*t for each sample and the Δ*C*t of empty vector transfected fibroblasts as control. The reported fold expression, expressed as Relative Quantity, was calculated by 2^−ΔΔ*C*t^. 

### 4.9. Statistical Analysis

Statistical comparison was performed using Student’s *t*-test. Differences were considered statistically significant when *p* < 0.05. For lipid analysis, 6 independent experiments for cells and EVs were carried out.

## Figures and Tables

**Table 1:** SFA, MUFA and PUFA content in control and H-RasV12 expressing fibroblasts and their released EVs. Data are expressed as amount of fatty acids relative to protein content (ng fatty acids/μg proteins). Mean values ± SD are shown (*n* = 6) (* *p* < 0.05, control vs. H-RasV12). SFA: saturated fatty acids; MUFA: mono-unsaturated fatty acids; PUFA: poly-unsaturated fatty acids.

ng Lipid/µg Protein	Fibroblasts		EVs	
	CTRL	RasV12	CTRL	RasV12
**SFA**	212.39 ± 66.30	126.19 ± 47.05 *	1084.78 ± 147.63	787.4 ± 65.65 *
**MUFA**	35.14 ± 12.29	70.60 ± 31.65 *	40.38 ± 8.19	32.28 ± 7.22
**PUFA**	3.35 ± 1.54	3.38 ± 1.48	18.35 ± 8.46	11.67 ± 4.19

## Supplementary Materials

Supplementary materials can be found at http://www.mdpi.com/1422-0067/19/11/3515/s1.
