# Supplementary material for: Oncogenic H-Ras Expression Induces Fatty Acid Profile Changes in Human Fibroblasts and Extracellular Vesicles
Source: Int J Mol Sci. 2018 Nov 8;19(11):3515. doi: 10.3390/ijms19113515 (PMC6275056; doi:10.3390/ijms19113515)
Supplement: Supplementary file 1 [file ijms-19-03515-s001.pdf]

	Symbol	Entry	Forward	Reverse
**Desaturation**				
	**SCD**	NM_005063.4	TTCCCGACGTGGCTTTTTCT	AGCCAGGTTTGTAGTACCTCC
	**SCD5**	NM_001037582.2	ACTCTGCTCTGGGCCTACTT	GAGTACTTGTGGTGGGCTCG
	FADS1	*NM_013402.4*	*CAGCTATGGCCCCCGAC*	*ACAAAGGGATCCGTGGCAT*
	**FADS2**	NM_004265.3	CAGTCGGCAGGCAGCAT	GGAAGGCATCCGTTGCATCT
	**FADS3**	NM_021727.4	AGGACTCGTGCGTGCAG	GAAGGCATCCGTGGCGT
**Elongation**				
	**ELOVL1**	NM_001256402.1	TGAAGCACGCAGTTCCTGAT	GGAGAAGAGGAAGAGCCAGG
	ELOVL2	*NM_017770.3*	*CGCTGCGGATCATGGAACAT*	*GTCCAACATGAACCACCCTCT*
	ELOVL3	*NM_152310.2*	*ACCTCATTCCCCATAGCCCT*	*TGCCCCCAGGATACTGAAGA*
	ELOVL4	*NM_022726.3*	*AGTTCTACCGCTGGACCTG*	*TGATCCCATGAATAACTCTCTGAA*
	**ELOVL5**	NM_021814.4	GCGCTTGATTCATCCTTCGG	CCATTTGAAAACCTTTTAGCCCA
	**ELOVL6**	NM_001130721.1	AAAGCACCCGAACTAGGAGA	GCCCGCAAGGCATAGTAAGA
	ELOVL7	*NM_024930.2*	*AGATGCTGATCCAAGAGTTGAAG*	*TGAGTTCAAAGGGCTTGCGA*
**Conjugation of long acyl chain fatty acids to acyl CoA**				
	ACSL1	*NM_001286710.1*	*GAGCTTTTGCAGCACTCACC*	*ACAAGGGCCATTATTTGACACC*
	**ACSL3**	NM_004457	ATTGTGCATACCATGGCTGCAGTG	TCTGGAATCCTTTCTGCCATCCCA
	**ACSL4**	NM_004458	TGGGCATTCCTCCAAGTAGACCAA	ACTGGCCTGTCATTCCAGCTATCA
	**ACSL5**	NM_016234	TCAGTCATCACATTCTTCCGGGCA	CCAGCTTCACGTAATTGCAAGCCA
	ACSL6	*NM_015256*	*AGCTGGCCTGCTACACATATTCCA*	*TCCACATGCTCTAGCAGAAGCACA*
**Phospholipases**				
	PLA2G2A	*NM_001161729.1*	*ATCACCTGTGCAAAACAGGACT*	*TTCCAGGGAAGAGGGGACTC*
	PLA2G2C	*NM_001316722.1*	*TGACACTGACAGCCCCTCAT*	*ACAAACCACTGCGCCATTGA*
	PLA2G2D	*NM_012400.3*	*GTGGTGATGGCTGGTGTGAT*	*CACCTAGTCCGCAGTGACAG*
	PLA2G2E	*NM_014589.2*	*TATGGCTGTTACTGCGGCAT*	*CTGCCGGCGCAGAAAATG*
	PLA2G2F	*NM_022819.3*	*GGATGAGGTGGACTGGTGC*	*TCCTCTCGGTACGTCTGGTT*
	**PLA2G3**	NM_015715.4	CTCGGAGCTGGGGGTCTT	GGGATCTCCAGCACGTTGAA
	**PLA2G4A**	NM_024420	TGAGTGACTTTGCCACACAGGACT	AATGTGAGCCCACTGTCCACTACA
	**PLA2G4B**	NM_001114633	TGGGAACTGTCATCACGC	AAGTGCTCCAACTACTCAACGT
	PLA2G4C	*NM_003706*	*CTGGTGGATGCTGGTTTAG*	*GCGGCAGTAGTCAGTGGTA*
	**PLA2G4D**	NM_178034.3	GCTGACCTGTTGAGTGAGGC	ATCCTCCGTGACTGAGTCCT
	PLA2G4E	*NM_001206670.1*	*AGCCCACAAACGGATGAAGA*	*TCTGGCTCAGCATATCAGCCT*
	PLA2G4F	*NM_213600.3*	*TGCTGTGAAGAACGTCCTGG*	*ACCCCGTTGGTGATGACTTC*
	PLA2G5	*NM_000929.2*	*CGGTCACTCCCATTCACAGC*	*CTCTGGGGTTCTCTTTGGCCT*
	PLA2G6A/ *PNLPA9*	*NM_003560*	*GAGAGCACGGCAACAC*	*GGAGGCTAGGAATGTA*
	**PLA2G6B/** PNLPA8	NM_015723	TCATCTGCTGCTCCAGGCTACTTT	CGGCACATCTGGCCAAAGACATTT
	PLA2G7	*NM_001168357.1*	*GTCTTGGGGCATTCAGGACA*	*TGCCGTACCTGCTCATTTCG*
	**PLA2G12A**	NM_030821.4	TGCAGTGACGGATCTAAGCC	TGCTTTTGCCACAGGTCTCA
	PLA2G12B	*NM_032562.4*	*TGCCGATATGGAAAGGCACC*	*ATTTGTTGGCACCGCAAGTG*
	**PLA2G15**	NM_012320.3	CTGGTCCCTGGTGATTTGGG	AACCAGCCTGATATTGTCAATCC
	**PLA2G16**	NM_001128203.1	GCCCTGCAGCAAAATCATCC	CGATGATGACATCTCTGACCTGG

Supplementary Table 1. Primers used for qRT-PCR. The symbols used in the main text are indicated in bold. Italics indicates genes that are not expressed in HuDe fibroblasts.
